# CMTX1 patients’ cells present genomic instability corrected by CamKII inhibitors

**DOI:** 10.1186/s13023-015-0270-5

**Published:** 2015-05-07

**Authors:** Mones Saleh, Gess Burkhardt, Bordignon Benoit, Altié Alexandre, Young Peter, Bihel Frederic, Fraterno Marc, Peiretti Franck, Fontes Michel

**Affiliations:** NORT. UMR INSERM 1062, INRA 1260, Aix Marseille Université, Campus Santé La Timone, 27 boulevard Jean Moulin, Marseille, 13385 Cedex 53 France; Department of Sleep Medicine and Neuromuscular Disorders, University Hospital Muenster, Muenster, Germany; Department of Neurology, Aachen RWTH University Clinic, Aachen, Germany; Service de Microscopie Electronique, Faculté de Médecine de la Timone, 27 boulevard Jean Moulin, Marseille, 13385 Cedex 53 France; Laboratoire d’Innovation thérapeutique, UMR7200, CNRS, Université de Strasbourg, Faculté de Pharmacie, 74, route du rhin, Illkirch Graffenstaden, 67400 France

**Keywords:** CMT disorder, Peripheral neuropathies, Drug development

## Abstract

**Background:**

We previously described that fibroblasts from animal models of CMTX1 present genomic instability and poor connexon activity. In vivo, these transgenic mice present motor deficits. This phenotype could be significantly reverted by treatment with (CamKII) inhibitors. The objective of this study is to translate our findings to patients.

**Methods:**

We cultured fibroblasts from skin biopsies of CMTX1 patients and analyzed cells for genomic instabilty, connexon activity, and potential correction by CamKII inhibitors.

**Results:**

The phenotypic analysis of these cells confirmed strong similarities between the *GJB1* transgenic mouse cell lines and CMTX1 patient fibroblast cell lines. Both present mitotic anomalies, centrosome overduplication, and connexon activity deficit. This phenotype is corrected by CamKII inhibitors.

**Conclusions:**

Our data demonstrate that fibroblasts from CMTX1 patients present a phenotype similar to transgenic lines that can be corrected by CamKII inhibitors. This presents a track to develop therapeutic strategies for CMTX1 treatment.

## Background

We recently created and analyzed transgenic mouse lines that expressed a human mutated GJB1 [1] (i.e., the gene involved in the X-linked form of Charcot-Marie-Tooth disease [2]), coding for connexin 32 (Cx32), a component of gap junctions [3,4]. We demonstrated that Cx32 is involved in mitotic stability, as transgenic cells present mitotic instability (i.e., aneuploidy, or centrosome overduplication). Both our study and the European Mitocheck project (www.mitocheck.org) observed that a lower Cx32 expression or expression of a mutated isoform resulted in perturbation of cell division [1,5].

Moreover, in a recent article [6], we suggested that this instability is due to CamKII overexpression, thereby leading to centrosome overduplication. In 1997, Torok et al. [7] identified two calmodulin-binding domains in Cx32 and provided evidence that calmodulin may function as an intracellular ligand, regulating Ca^2+^-dependent intercellular communication across gap junctions. Finally Dodd et al. [8] demonstrated that the physical proximity between Cx32 and Calmodulin Kinase II (CamKII) had a physiological role. It was thus likely that pathological mutations in Cx32, associated to CMTX1, resulted in mitotic instability through CamKII overexpression that led to centrosome overduplication. Furthermore, we demonstrated that treatment with CamKII inhibitors [6] (KN62 or KN93 [9]) resulted in a partial but significant rescue of abnormal centrosome overduplication, mitotic instability, and connexon activity. In addition, in vivo treatment of CMTX1-related transgenic mice with KN93 improved their locomotor performance on the rotarod.

However, these data were either obtained from transgenic mouse lines (i.e., our findings) or human transfected cells (i.e., the Mitocheck project). We could thus not presume that these findings could be translated to CMTX1 patients. We thus collected and analyzed fibroblasts from the skin biopsies of five CMTX1 patients that presented three different mutations. Phenotypic analysis of the patient’s cells were compared to phenotypic presentation of the cells from CMTX animal models. Phenotypic correction using CamKII inhibitors was also tested.

## Methods

### Patients

Patients were evaluated at the University Hospital of Muenster. The inclusion criteria for this study required a clinical phenotype consistent with CMT and a genetic diagnosis of CMTX1. Nerve conduction studies (NCS) were performed according to standard procedures.

### Standard protocol approvals, registrations, and patient consents

All biological materials family history as well as medical and neurophysiological reports were obtained under appropriate informed consent of the patients or their legal guardians. The local ethics committees of the University of Muenster approved the study.

### Mutations and clinical presentation of patients

**Patient 1** is a female with the mutation R215W on the gene *Gjb1*. Clinically, she has distal pareses of arms and legs, gait ataxia and distal sensory loss. Age of onset: 22 years. Nerve conduction studies showed an intermediate neuropathy.

**Patient 2** is the sister of patient 1, with the same mutation R215W. Clinically, she shows pareses of foot dorsal extension, finger abduction and extension on both sides, distal symmetric sensory loss and absent achilles tendon reflexes. Age of onset: 25 years.

**Patient 3** is a male with a mutation R142W. He presents muscle atrophies and pareses of hands and calves, foot drop, distal symmetrical hyp- and dysesthesia of legs. Tendon reflexes are reduced in the arms, absent in legs. Age of onset: ca. 15 years. Nerve conduction studies showed an intermediate neuropathy.

**Patient 4** is a female with the mutation V181M. She presents pareses of foot dorsiflexion and small hand muscles, neuropathic pain in feet and hands, distal symmetric hyp- and dysesthesia of feet and fingertips. Tendon reflexes reduced in the arms, absent in legs. Age of onset: ca. 25 years. Nerve conduction studies showed an intermediate neuropathy.

**Patient 5** is a male, the son of patient 4 with the same mutation V181M. He presents pareses of foot dorsiflexion, plantar flexion, mild pareses of finger abduction and hypesthesia of feet. He shows high arched feet and hammer toes. Tendon reflexes are absent in arms and legs. Age of onset: 16 years.

Results of nerve conduction studies are presented in Table 1.

**Table 1:** **Results of nerve conductions studies**

	**mNCV**	**CMAP**
Patient 1	39 m/s	3.4 mV
Patient 2	45 m/s	4.0 mV
Patient 3	32 m/s	1.5 mV
Patient 4	32.4 m/s	5.1 mV
Patient 5	34.1 m/s	1.4 mV

Electrophysiological evaluations were performed on 5 patients with CMTX1, with clinical presentations described in materials and methods. Results of nerve conduction studies in terms of median nerve motor conduction velocity (NCV) and compound muscle action potential amplitudes (CMAP) are presented in the table.

### Human fibroblast cell culture

Fibroblast cell cultures were performed as described [10]. A 1 mm skin punch biopsy was transferred to DMEM supplemented with 20% fetal calf serum, penicillin/streptomycin and glutamine (D20 medium). The tissue was transferred to a 60 mm plastic Pasteur dish and submersed in D20 medium at standard conditions. After a lawn of fibroblasts had grown, biopsy tissues were removed and the cultures were grown and passaged 3 times before assays were performed.

### Western blotting

Cells were lysed in RIPA buffer (50 mM Tris-Cl pH 7.4, 1% NP40, 0.25% sodium deoxycholate, 0.1% SDS, 150 mM sodium chloride) supplemented with protease and phosphatase inhibitors. The same amounts of protein from each sample were resolved under denaturing and reducing conditions on 4-12% NuPAGE gels (Invitrogen) and transferred to polyvinylidene fluoride membranes. Immunoreactive proteins were revealed by enhanced chemiluminescence with ECL (Perkin-Elmer). An antibody against phosphorylated CamKII (Cell Signaling, catalog number: 3361) was used.

### Centrosome labelling

Cells were grown on glass coverslips for 24 h to allow cultures to reach 80% confluence. To measure the number of centrosomes, cells were fixed with 4% PFA, permeabilized with methanol at −20°C for 8 min and blocked with 0.5% Triton X-100 in PBS for 30 min at RT. To detect γ-tubulin, cells were incubated overnight at 4°C with a mouse anti-γ**-**tubulin antibody (GTU-88; Sigma) diluted 1/1000 in PBS containing 0.1% milk and 0.05% Triton X-100. After washing, the cells were incubated for 1 h at RT with Cy3-conjugated goat anti-mouse IgG secondary antibody (Caltag Laboratories) diluted 1/2000 in PBS containing 0.1% milk and 0.05% Triton X-100. The preparations were counterstained with DAPI in Vectashield mounting medium (Vector Laboratories). Fluorescent images were acquired with a microscope (Leica DMR) equipped with a PL APO objective.

### Connexon activity

One hundred thousand cells were cultured as described above for one day with or without CamKII inhibitors (KN62 or KN93 at a final concentration of 10 μM). Lucifer yellow (LY) was added to the medium (final concentration: 110 μM) and incubated for two hours. Fluorescence was recorded using a Perkin Elmer Victor 4 microplaque reader (excitation: 405 nM, emission: 535 nM).

### Statistics

Statistical analysis was performed using Prism v5.0. Mann–Whitney and chi-square tests were used for trend analysis. The significance threshold was setted at p < 0.05.

## Results and discussion

### Nuclear anomalies

**Figure 1:**
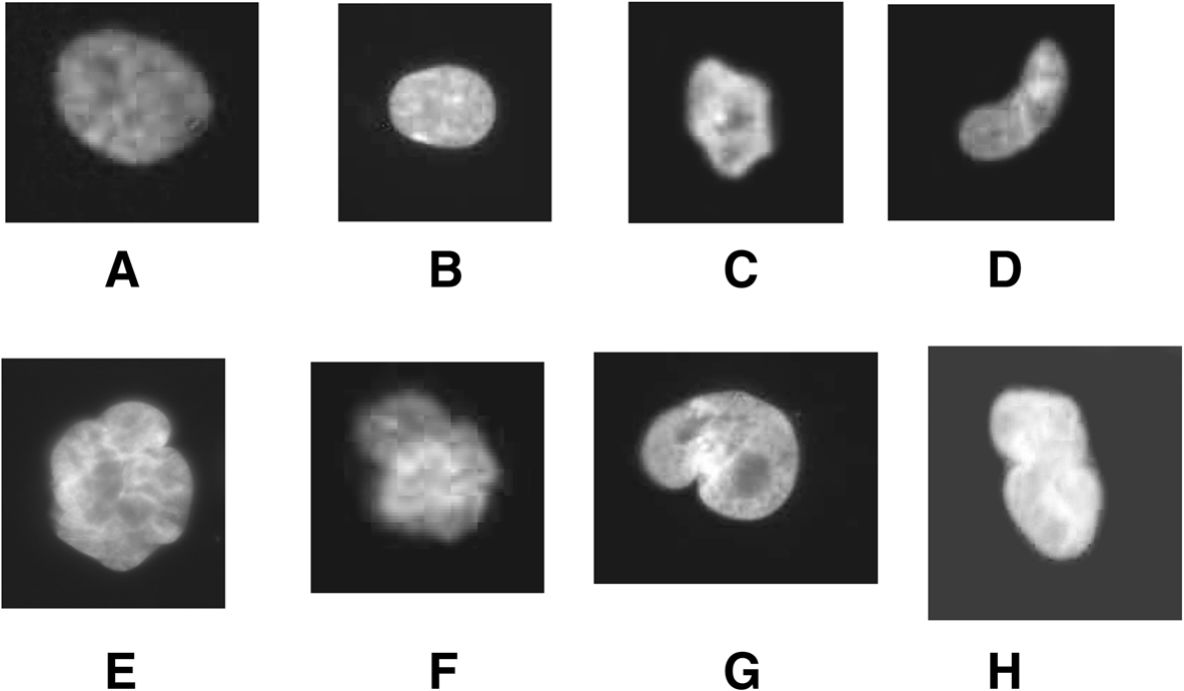
Patients fibroblasts have been cultured as described in methods. Nuclei have been stained with DAPI and captured using ligth microscope. Examples of abnormal nuclei observed in cells of CMTX1 patients. Normal nuclei **(A**, **B)**, Abnormal shape **(C and D)**, Polylobbed **(E and F)**. Non disjunction (**G** and marc).

**Figure 2:**
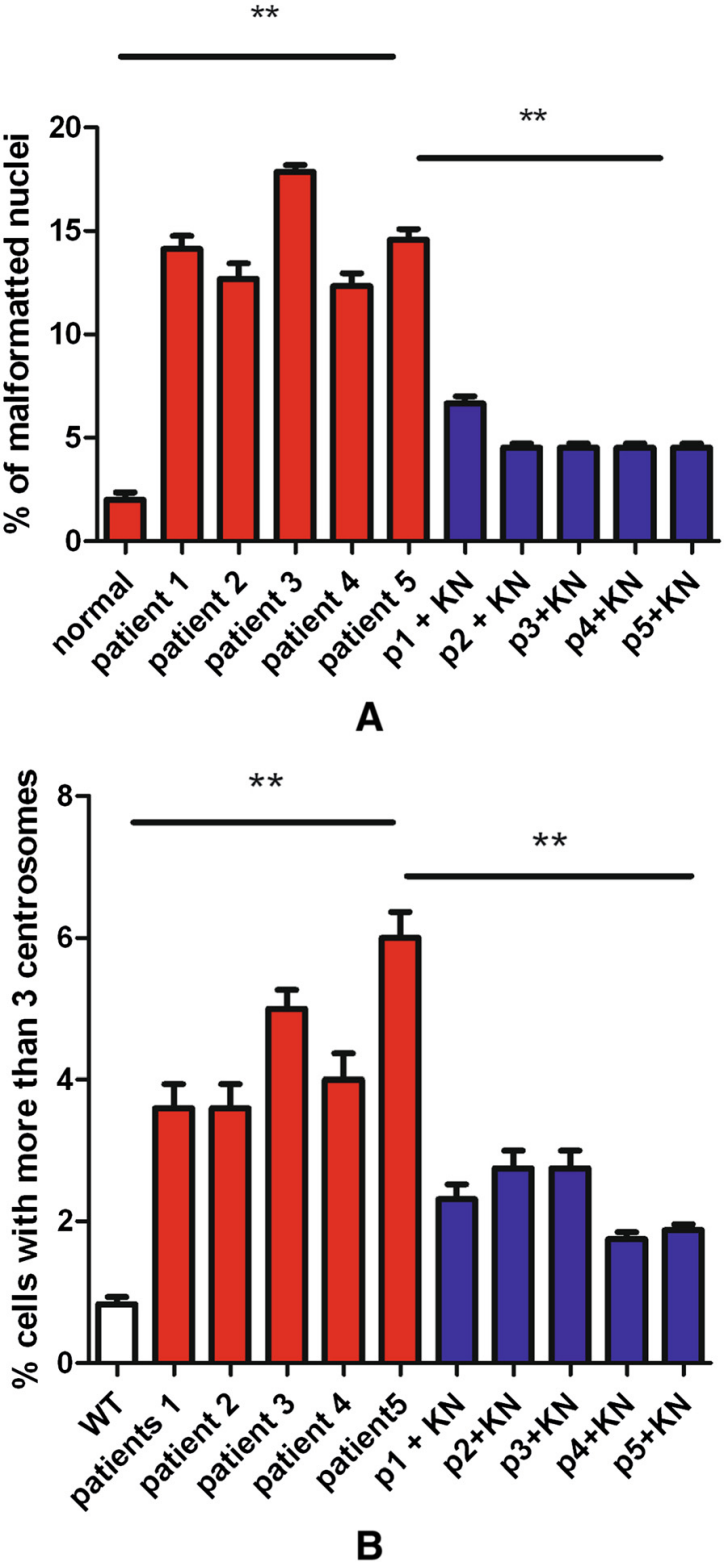
Number of nuclei with anomalies **(A)** and percentage of cells with an abnormal number of centrosomes **(B)** has been evaluated in cells of patients without or with treatment with an inhibitor of CamKII (KN93).

**Figure 3:**
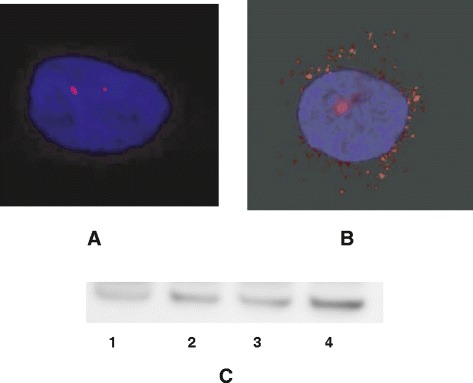
Patients fibroblasts have been cultured and centrosomes stained as described in Methods. Pictured have been captured using a fluorescence microscope. Examples are presented in Figure 3 **A** and **B**. Same cells have been lyzed, and analyzed usinh polyacrylamide gels. Western blats have been performed and probed using an antibody raised against the phosporylated form of CamKII (2C). 1, normal cells ; 2, cells from patient 1 ; 3, cells from patient 3 ; cells from patient 5.

As Schwann cell cultures could not be obtained from patients for practical and ethical reasons, we isolated fibroblasts from skin biopsies. Various parameters were analyzed. Patient fibroblasts did not show significant levels of polyploidy (not shown). However, we observed an abnormal number of nuclei presenting anomalies (Figure 1). Nuclear anomalies have been described and classified by the Mitocheck project: abnormal shape (C,D), polylobbed (E,F), two unsepared nuclei (G,F) and others [5]. We next quantified the percentage of these abnormal nuclei in CMTX1 patient fibroblasts, and compared the results to human fibroblasts from individuals that did not present CMTX1. We observed only a few abnormal nuclei in reference individuals (i.e., less than 3%), whereas approximately 15% of nuclei from CMTX1 patients had anomalies (Figure 2A). We have previously showed that CamKII activity was increased in transgenic cells and that inhibitors were able to reduced anomalies in *GJB1* transgenic cell lines. We thus evaluated CamKII acitvity in patient fibroblasts, using an antibody raised against phosphorylated CamKII, in patients fibroblasts. We could observed, in Figure 3C, that CamKII acitivty is overstimulated in patient fibroblasts (Figure 3C). According to these observations, fibroblasts from CMTX1 patients were treated in vitro with the CamKII inhibitor KN93 at a concentration of 10 μM. We found that KN93 was able to significantly reduce the amount of abnormal nuclei in fibroblasts from each CMTX1 patient, which supports our previous work on transgenic mice, (Figure 2A).

### Centrosome overduplication

Cells from five transgenic lines created in the laboratory present centrosome overduplications that are linked to mutations in *GJB1* [6]. We thus evaluated centrosome duplication in normal and CMTX1 fibroblasts, treated or untreated with the CamKII inhibitor KN93. We observed centrosome overduplication in the fibroblasts from CMTX1 patients, which supports the findings of the study on *GJB1* transgenic mice (Figures 3A, B, and 2A). As expected, this overduplication was significantly corrected by KN93 treatment (10 μM ; Figure 2B).

### Connexon activity

**Figure 4:**
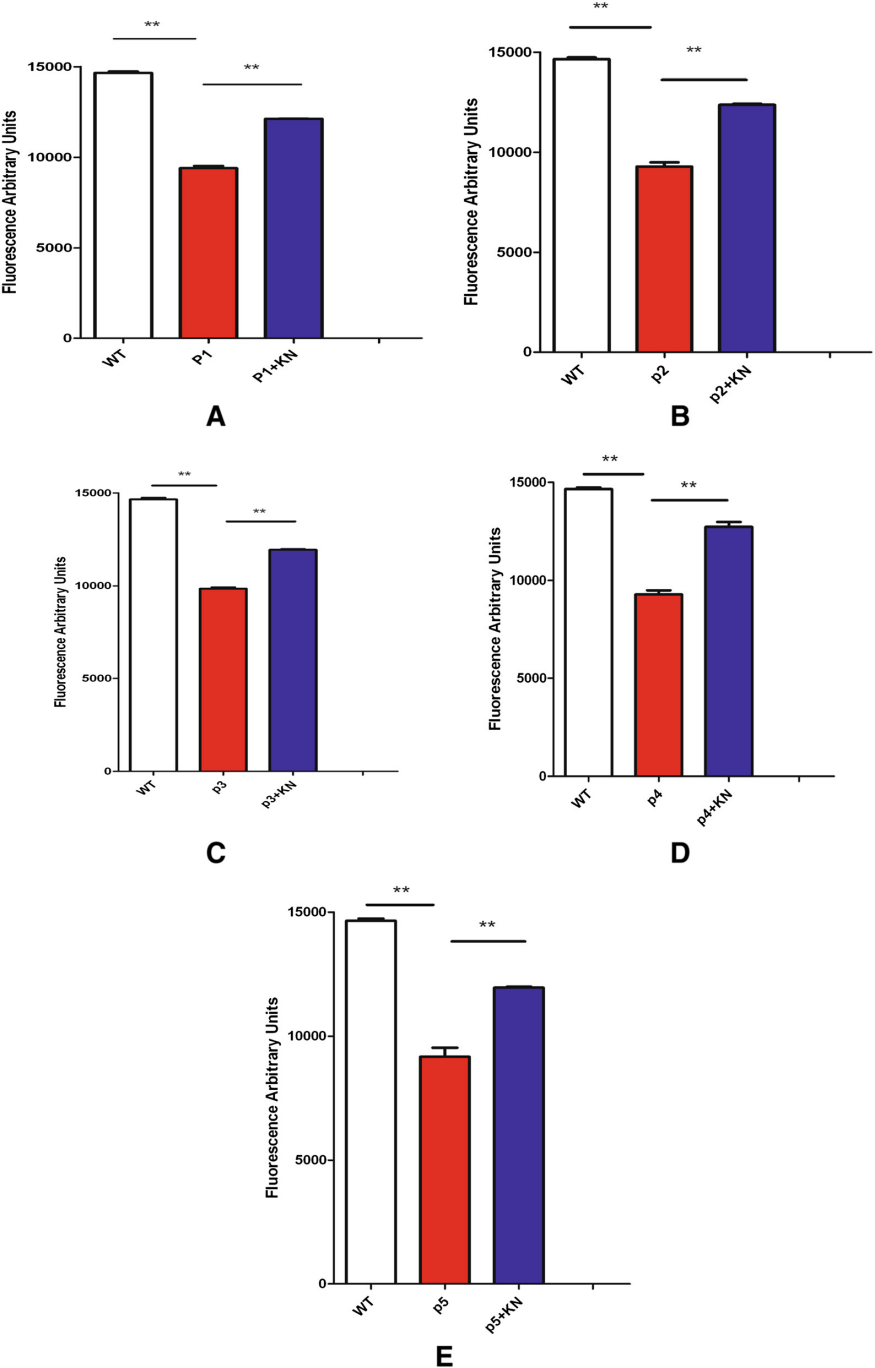
Connexon activity of patients cells (patient 1 to 5, **A**, **B**, **C**, **D** and **E**), and control human fibroblasts, has been evaluated using internalisation of Lucifer Yellow (LY). Fluorescence of LY has been recorded corresponding to cells treated or not with KN93.

Impairment of connexon activity is considered the primary cause of the CMTX1 phenotype in humans [11]. We thus evaluated the connexon activity of the fibroblasts from CMTX1 patients, using an assay developed in our laboratory [6] which is based on the measurement of Lucifer Yellow internalization that requires connexon activity. Connexon activity was found to be lower in CMTX1 patient fibroblasts as compared to healthy controls (Figure 4). After treatment with KN93, the connexon activity significantly improved in the fibroblasts of each CMTX1 patient (Figure 4).

## Conclusions

In conclusion, the fibroblasts from five CMTX1 patients showed the same cellular phenotype that we described in *GJB1* transgenic mouse models created in the laboratory [1,6], including nuclei anomalies, centrosome overduplication, and impaired connexon activity. As suggested by Matsumoto and Maller [12], centrosome duplication is linked to CamKII activity. In CMTX1 mice, we have already shown that CamKII inhibitors can revert the phenotype linked to mutations in the *GJB1* gene. These results suggest that the phenotype observed in the fibroblasts from CMTX1 patients can also be corrected, at least partially, by treatment with a CamKII inhibitor.

Waggener et al. recently demonstrated that CamKII is involved in myelination mechanisms in the central nervous system (CNS) [13]. They demonstrated that perturbation of CamKII beta is associated with anomalies in CNS glial celll maturation, is involved in anomalies of actin skeleton, and is associated with myelin anomalies. Recently, we demonstrated that the locomotor behaviour of *GJB1* mutated mouse models of CMTX1 can be improved by treatment with CamKII inhibitors [6]. In conclusion, the fibroblasts of human CMTX1 patients present the same phenotype as the fibroblasts of mouse models. Moreover, the same molecule (KN93) partially corrects the cellular phenotype of human and mouse fibroblasts as well as locomotor behaviour in mouse models. These findings provide a translational link from the murine to the human system. Although it is still too early to directly apply our results to human patients, for the first time, our results show a potential avenue for therapeutic approaches to CMTX1 treatment.
