# Erratum to: CMTX1 patients’ cells present genomic instability corrected by CamKII inhibitors

**DOI:** 10.1186/s13023-015-0354-2

**Published:** 2016-01-25

**Authors:** Saleh Mones, Burkhard Gess, Benoit Bordignon, Alexandre Altié, Peter Young, Frederic Bihel, Marc Fraterno, Franck Peiretti, Michel Fontes

**Affiliations:** NORT. UMR INSERM 1062, INRA 1260, Aix Marseille Université, Campus Santé La Timone, 27 boulevard Jean Moulin, Marseille, 13385 Cedex 53 France; Department of Sleep Medicine and Neuromuscular Disorders, University Hospital Muenster, Muenster, Germany; Department of Neurology, Aachen RWTH University Clinic, Aachen, Germany; Service de Microscopie Electronique, Faculté de Médecine de la Timone, 27 boulevard Jean Moulin, Marseille, 13385 Cedex 53 France; Laboratoire d’Innovation thérapeutique, UMR7200, CNRS, Université de Strasbourg, Faculté de Pharmacie, 74, route du rhin, Illkirch Graffenstaden, 67400 France

## Erratum

After publication of [1] it came to the authors’ attention that all author names were incorrect. The correct spellings of their names have been included in this erratum.
